# Charge Splitting In Situ Recorder (CSIR) for Real-Time Examination of Plasma Charging Effect in FinFET BEOL Processes

**DOI:** 10.1186/s11671-017-2309-0

**Published:** 2017-09-18

**Authors:** Yi-Pei Tsai, Ting-Huan Hsieh, Chrong Jung Lin, Ya-Chin King

**Affiliations:** National Tsing Hua University, Hsinchu, Taiwan

**Keywords:** Plasma-induced damage, Advanced FinFET technology, Charge splitting

## Abstract

A novel device for monitoring plasma-induced damage in the back-end-of-line (BEOL) process with charge splitting capability is first-time proposed and demonstrated. This novel charge splitting in situ recorder (CSIR) can independently trace the amount and polarity of plasma charging effects during the manufacturing process of advanced fin field-effect transistor (FinFET) circuits. Not only does it reveal the real-time and in situ plasma charging levels on the antennas, but it also separates positive and negative charging effect and provides two independent readings. As CMOS technologies push for finer metal lines in the future, the new charge separation scheme provides a powerful tool for BEOL process optimization and further device reliability improvements.

## Background

Plasma-enhanced processes are widely used in the formation of fin field-effect transistor (FinFET) circuits, which composed of many high-aspect ratio structures and fine metal lines [1]. During manufacturing, etching and deposition step for realizing these 3D compositions can lead to significant plasma-induced stress to the FinFET devices [2–4]. As CMOS FinFET technology advances, the metal linewidth and pitch reduce more aggressively than its height, driving the need for forming high aspect ratio trenches defined by extremely fine lines. Unavoidably, this promotes the severity for plasma-induced damage (PID) to the transistors, and its corresponding effect on circuit reliability becomes one of the key concerns in developing FinFET technologies [5–7]. In forming small contacts, vias, and fine metal lines, strong power and high-selectivity plasma are generally applied [8]. Moreover, in etching the bulk fin, sputtering of reactive ions on the fin surface can lead to defects in the bulk fin, critical to the characteristics of the transistors [9]. In order to enable fin metal gate and dense interconnect structures, complex metal stacks are more often used in advanced FinFET technologies [10, 11]. In addition, high-*k* gate dielectric used in advanced technology usually leads to enhanced stress-induced trapping after plasma process [12–14]. During plasma charging, discharging path through narrow fins and to the substrate can lead to more non-uniform stress levels across a whole wafer [15]. The plasma-induced stress on the transistor gate oxide is known to result in the further degradation of the gate dielectric integrity [16, 17].

The plasma-induced damage on gate dielectric film can lead to performance degradation in highly non-uniform charging scenarios, even yield loss [18–20], as a result of reliability failures [21, 22]. Thus, in advanced FinFET technologies, test devices with enlarged antenna structures are generally used for monitoring PID effects, which provide feedbacks for further process optimization.

The most common and widely used measure of PID is the time-to-breakdown characteristic of the test samples with large antenna structures. The latent damage on these PID patterns are typically reflected by measuring the time-dependent degradation of the gate dielectric layers, hence, failing to give the real-time feedback of the plasma processes [23]. In addition, conventional test devices cannot tell the sources and the polarity of plasma-induced charging rate and or maximum potential build-up on the antenna. A PID recorder with a floating gate coupled by antenna structure has been proposed with in situ detection capability in our previous study [24, 25]. In this work, we proposed a revised PID recorder with a charge splitting feature. Through a forward diode and a reverse diode connected to a common antenna structure, the new design provides separate paths for positive and negative charges. Therefore, charging levels of both polarities can be independently recorded. This new charge splitting in situ recorder (CSIR) requiring only small antennas enables future study of plasma charging effect in middle-end of the line (MEOL) processes.

## Methods

### Plasma Charging Polarity

**Fig. 1:**
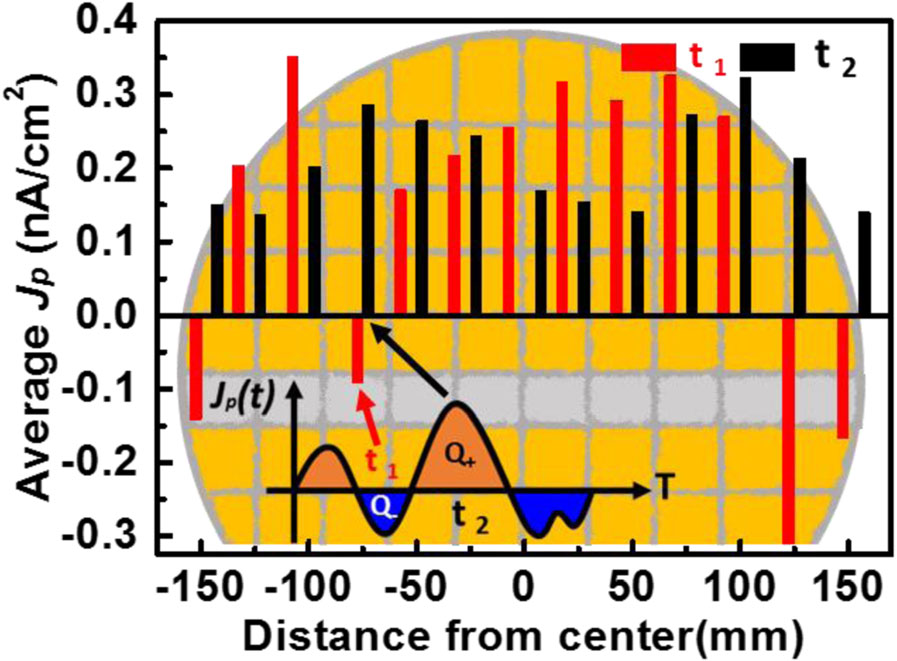
Distribution of plasma-induced charging rate in the center line of the wafer during etching process at different times. The plasma charging polarity on a particular location may change over time

Previous studies reported that, during etching process in forming poly- or metal layers, the plasma inhomogeneity as well as the variations of the antenna potential can lead to a drastic difference in the charging rate or even the polarities may change at different locations [24, 25]. The macro-environment in the plasma chamber and micro-patterns can both affect the distribution of charging rates on a wafer [26]. Namely, plasma charging rate in back-end-of-line (BEOL) etching varies spatially and timely. During radio frequency (RF) plasma processes, the surface of the wafer collects charging current, *J*_p_, which is composed of an ion current *J*_i_ and an electron current *J*_e_ [26]. The ion current is almost constant with time and is determined by the ion density *J*_i_ and the Bohm velocity [26]. Since the plasma potential *V*_p_(*t*) is higher than the gate potential *V*_G_ for most of the time, the electron current flows only during the short periods when the plasma potential is near its minimum. During *Q*_FG_ process, the gate voltage may increase or decrease over many RF cycles, depending on which component of the currents is larger, until a steady-state gate voltage is reached when the tunneling current balances *J*_*p*_ on the antenna. As shown in Fig. 1, the distribution of plasma charging rate, *J*_P_ (*x*,*y*,*t*), across the wafer during etching process at different stages changes in both magnitude as well as in polarities, where it can be expressed as in Eq. (1) where *J*_e_ represents electron current density, and *J*_i_ represents ion current density.

1 $$ {J}_{\mathrm{p}}={J}_{\mathrm{e}}+{J}_{\mathrm{i}}\dots $$

**Fig. 2:**
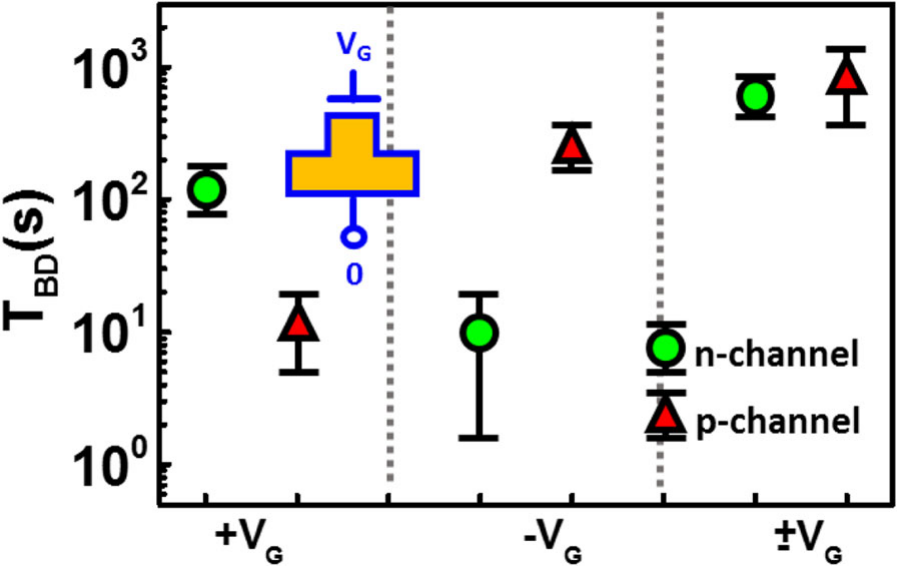
Time-to-breakdown (*T*_BD_) of n-channel and p-channel FinFETs stressed by positive, negative, and positive + negative charging on the gate electrodes. *T*_BD_ of devices under different polarity stresses suggests that the damage accumulated on the gate dielectric depends not only on the charging polarity, but also the wells under corresponding FinFETs

**Fig. 3:**
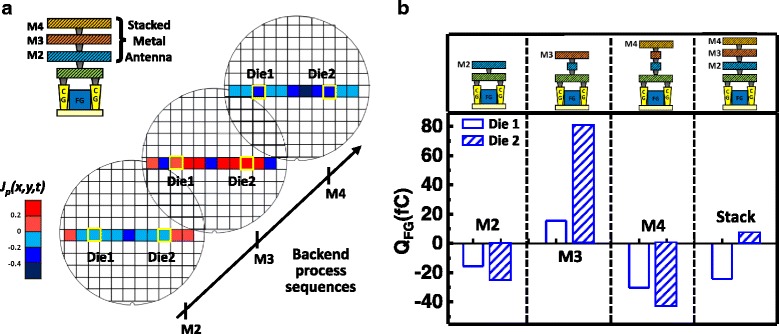
**a** The plasma charging effect for the different metal layers varies on different locations across the wafer. **b** The positive and negative charges may compensate each other in the stacked metal layers

The different plasma charging polarities result in either positive or negative antenna charge, *Q*_P_, accumulated at a different time and location. To clarify, at time *t*_1_, a negative *J*_p_ leads to negative antenna charge *Q*−. At *t*_2_, a positive *J*_p_ induces a positive antenna charge Q+ on the identical location on the wafer, as illustrated in Fig. 1. Thus, positive or negative charge may accumulate on a same antenna at different time during the etching process. From previous reports [27], the peak levels of *J*_e_ and *J*_i_ are around − 0.15 and 0.35 mA/cm^2^, respectively. It has been found [28, 29] that DC and AC/bi-directional gate stress on n-channel and p-channel FinFET results in different latent damage to the gate dielectric film. High voltage stresses with positive or negative DC bias and AC voltage with a switching frequency of 0.1 Hz are applied to conventional FinFET test samples, respectively. As shown in Fig. 2, the time-to-breakdown (*T*_BD_) of a transistor stressed by positive, negative, and gate stress in both directions are compared. The results indicate that DC gate stress will cause worse damage on the samples, while AC gate stress results in less severe damage to these transistors, as suggested by the longer *T*_BD_ for samples subjected to bi-directional stress. Figure 2 also shows that the oxide degradation depends not only on the charging polarity, but also on the type of wells under the n-channel and p-channel transistors, which is expected to be caused by the difference in the discharging paths of these test devices during process. Hence, conventional PID detector, which uses *T*_BD_ as the indicator for damage severity cannot reflect the plasma charging level during the process. On the other hand, the plasma charging recorder proposed in our previous work records the stress level by injecting or ejecting electron to/from a floating gate (FG) coupled by a charge-collecting antenna. The recorded data, floating gate charge (*Q*_FG_), is read subsequently after fabrication [24, 25]. The recording is then measured by threshold voltage shift on the read transistor, of which the channel is controlled and directed by the same floating gate. The raised potential on the antenna with *Q*_P_ from plasma charging can induce both positive and negative antenna voltage during the formation of a single metal layer. Further, for different metal layers, different manufacturing parameters are used. For example, etching time, chemical used, and chamber temperatures may vary. These parameters can affect the antenna charge distribution across a wafer during etching. In other cases, a transistor with connections to multiple metal layers subject to even more complex plasma charging sequences, as illustrated Fig. 3a.

At different stages of the BEOL process, the plasma charging current at a particular antenna can switch between ion and electron current, i.e., the net *Q*_P_ can also shift from positive to negative. The recordings on samples with antenna consisting of metal 2, metal 3, metal 4, and multiple metal layers are summarized in Fig. 3b. Data suggests net charging of a single metal layer [24] on a particular change polarity from layer to layers. In addition, the averaging effect found on the *Q*_FG_ of the samples with antenna structures of multiple metal layers is further supported by the measured data in Fig. 3b. With both positive and negative *V*_*G*_ on the antenna, the final *Q*_FG_ will then be averaged out by electron injection and ejection into/from the FG which may occur sequentially. This compensation effect will limit the recorder to reveal the real stress conditions a device experienced during plasma processes. The revised CSIR is designed to address the problem on how to individually record positive and negative charging effects without interference and to supply more detailed data on the charging situation in the plasma chamber.

### Test Pattern for Charge Separation

**Fig. 4:**
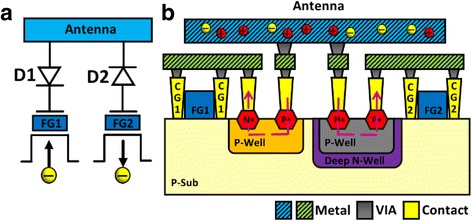
**a** Charge splitting in situ recorder with two separate floating gates by connecting to a forward diode (D1) and a reverse diode (D2) for detecting electron/ion charging, respectively. **b** Cross-sectional illustration of the new charge splitting in situ recorder with on-chip pn diodes, directing the positive and negative charge to the separated coupling gates, CG_1_ and CG_2_

In this research, the positive ion charging and negative electron charging on the antenna can be separated with a new charge splitting in situ recorder (CSIR) proposed, as illustrated in Fig. 4a. A CSIR consists of two floating gates, FG_1_ and FG_2_ which record the different types of charging effect separately. The antenna structure connects to the two coupling gates through a forward diode (D1) and a reverse diode (D2), respectively. In the left half of the structure, the positive charges will flow into the coupling gate 1 (CG_1_) through D1. When CG_1_ is positively charged, the voltage is coupled to the floating gate through the contact slots on both sides. The floating gate will be negatively charged as electrons inject from the substrate. The right half of the structure on the other hand is the negative charging path, allowing current to flow from the antenna into the coupling gate 2 (CG_2_) through D2, resulting in positive *Q*_FG_. Figure 4b further shows the cross-sectional view of the CSIR with on-chip pn diodes, directing the positive and negative charging paths to the separate coupling gates, CG_1_ and CG_2_, which couple the potential on the antenna to the FG_1_ and the FG_2_, respectively.

**Fig. 5:**
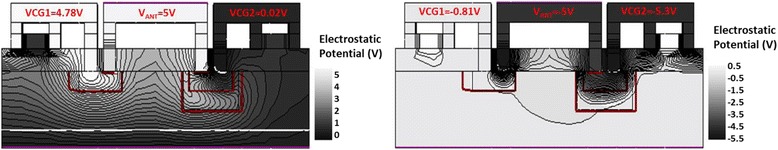
Simulated potential distribution in CSIR with positive and negative antenna gate voltage. The forward and reverse pn diodes successfully separate the antenna charge polarity

When the left half of the recorder are enabled in the CSIR under a positively charged antenna, the right half is inactive as charge is blocked by the reverse diode, and vice versa. Both on-chip diodes are composed of n+/p-well. For D2, to sustain negative voltage in its p-well, the p-region needs to be surrounded by a deep n-well, blocking the charging path directly to the substrate. The simulated potential distribution on the cross-section in a CSIR under positive and negative charging periods of the antenna is shown in Fig. 5a and b, respectively. Assuming that the potential on an antenna reaches 5 V, through the diode on the left, positive charge flows to the control gate on the left, which results in a high positive voltage (*V*_CG1_). At the same time, positive charge is blocked by the diode on the right, resulting in a close-to-zero *V*_CG2_. The difference in potential on the two control gates are verified by the simulated potential contours in Fig. 5a. The effect of negative charging on the antenna is shown in Fig. 5b. Simulated potential profiles verify that the on-chip pn diodes can effectively direct and block the potential to CG_1_ and CG_2_, complimentarily, as designed. This way, positive and negative charging effects corresponding to different sources in the plasma treatments can be independently obtained, preventing charge compensation and interference issues on the detector.

## Results and discussion

**Fig. 6:**
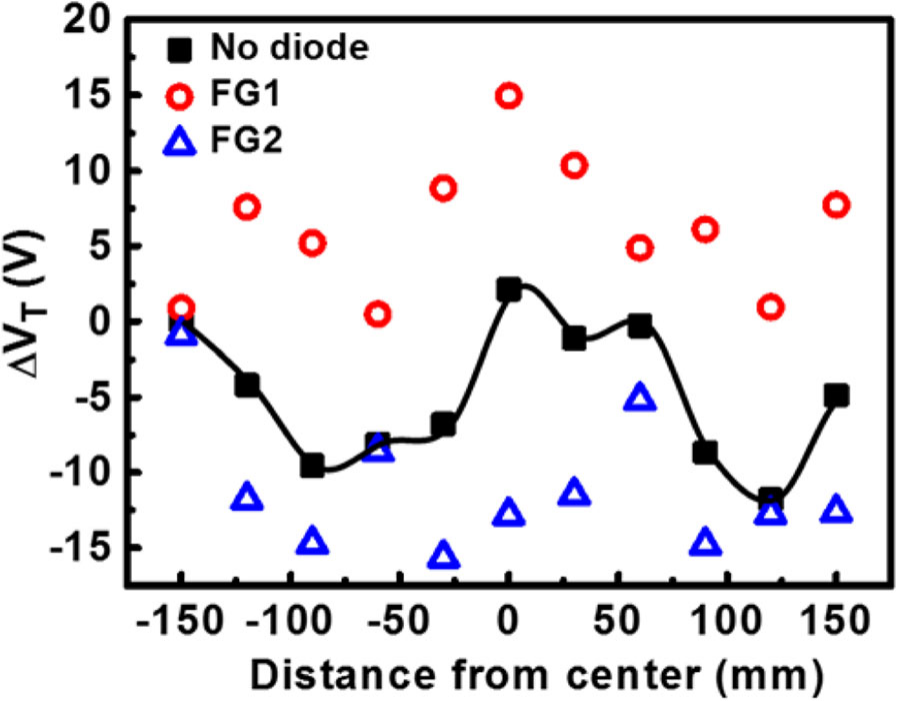
Distribution of delta *V*_T_ on FG_1_ with forward diode and FG_2_ with reverse diode, and FG without diode along the center line of a wafer

**Fig. 7:**
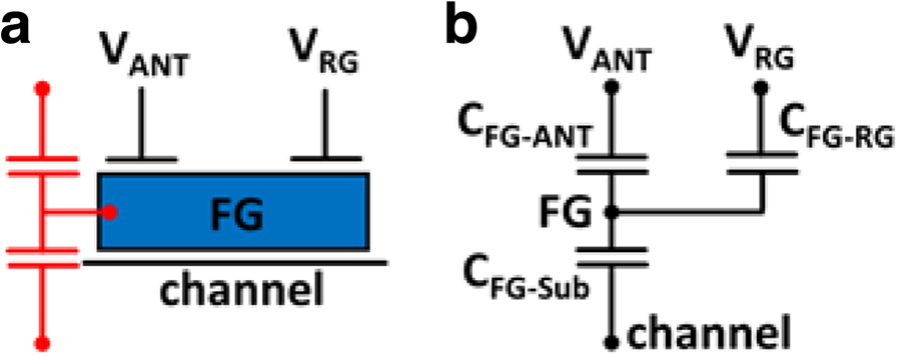
**a** The schematic diagram of a capacitance network model in a CSIR device. **b** The total capacitance of floating gate is all mentioned capacitances in series plus that in parallel

The measured threshold voltage shift (Δ*V*_T_) on device controlled by FG_1_ with forward diode and that by FG_2_ with reverse diode and samples without diode are compared in Fig. 6. Data along the center line of a wafer reveal that a recorder with a single floating gate does subject to charge neutralization even within the processing of a single metal layer. The averaging effect of a recorder without diode proves that the peak charging rates will be not reflected truthfully. On the other hand, readings from the new CSIR can provide positive and negative charging levels, independently. To further investigate the plasma charging effect in metal 2 (M2) formation, the collected charge on FG_1_ and FG_2_ of the CSIR of each dies can be independently calculated by Eq. (2),

2 $$ {Q}_{\mathrm{FG}}={C}_{\mathrm{T}}\times \Delta  {V}_{\mathrm{T}}\times {\alpha}_{\mathrm{RG}}\dots $$

where *Q*_FG_ is the charge in the floating gate. *C*_T_ is the total capacitance of the floating gate, as illustrated in Fig. 7. Δ*V*_T_ is the threshold voltage shift detected at the read gate of the recorder, while *α*_RG_is the coupling ratio from the read gate.

When floating gate charge is initially zero and *Q*_FG_ reaches the saturated level when the electric field across the gate dielectric layer is reduced to zero, the final antenna gate potential at the end of a plasma process can be expressed as followed,

3 $$ {V}_{\mathrm{ANT}}=\frac{V_{\mathrm{FB}}-\frac{Q_{\mathrm{FG}}}{C_{\mathrm{T}}}}{\alpha_{\mathrm{ANT}}}\dots $$

in which, *V*_ANT_ is antenna gate potential by plasma charging and *α*_ANT_ represents the coupling ratio to the floating gate from the antenna gate. *V*_FB_ is the flatband voltage from the metal gate to the fin-substrate. Under a given process time, the average plasma charging current density, *J*_p_ can be then projected by Eq. (4).

4 $$ {J}_{\mathrm{p}}=\frac{V_{\mathrm{ANT}}\times {C}_{\mathrm{ANT}}}{A_{\mathrm{ANT}}\times \Delta  t}\dots $$

where Δ*t* is the duration of a plasma process [28, 29] and *C*_ANT_ is the total capacitance of the metal antenna, while *A*_ANT_ is the charging area of an antenna. All the parameters used in the above calculations are summarized in Table 1.

**Table 1:** Parameters in Eqs. (2)–(4) for estimating plasma charging current density during BEOL processes

Parameters	Definition	Unit
*Q*_FG_	Floating gate charge	V
*C*_*T*_	Total capacitance of the floating gate	F
*ΔV*_*T*_	Threshold voltage shift	V
*α*_RG_	Coupling ratio from the read gate	
*V*_ANT_	Antenna gate potential	V
*V*_FG_	Floating gate potential	V
*V*_FB_	Flat band voltage	V
*α*_ANT_	Coupling ratio from antenna gate	
*J*_*p*_	Plasma charging current density	A/cm^2^
*Δt*	Plasma process duration	sec
*C*_ANT_	Capacitance of the metal antenna	F
*A*_ANT_	Area of the metal antenna	cm^2^

**Fig. 8:**
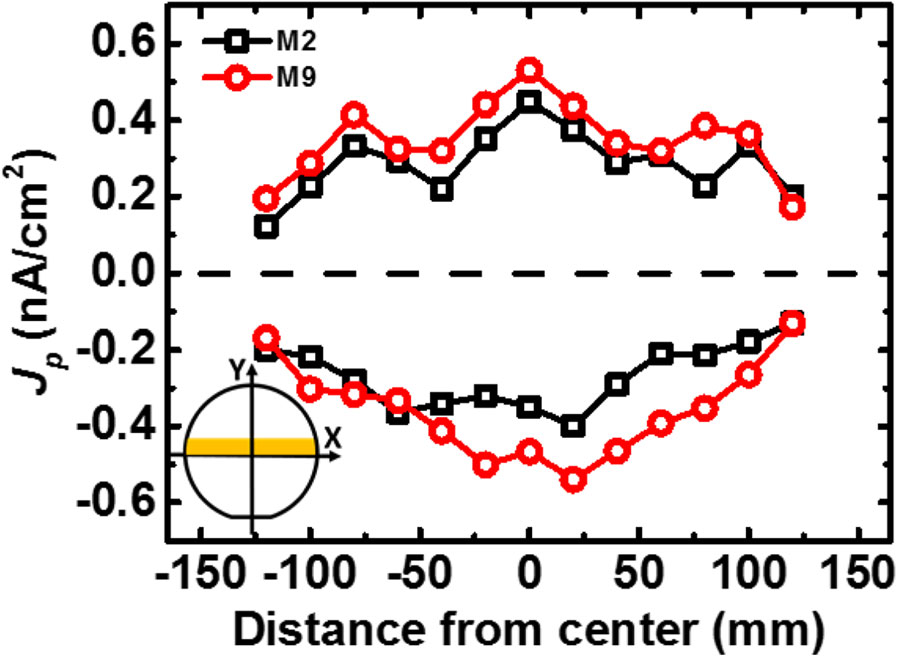
Comparison of positive and negative charging rate in the center line of a wafer for metal 2 and metal 9 processes. The charging rates peak around the center which means plasma-induced damage is more severe in the center of the wafer

**Fig. 9:**
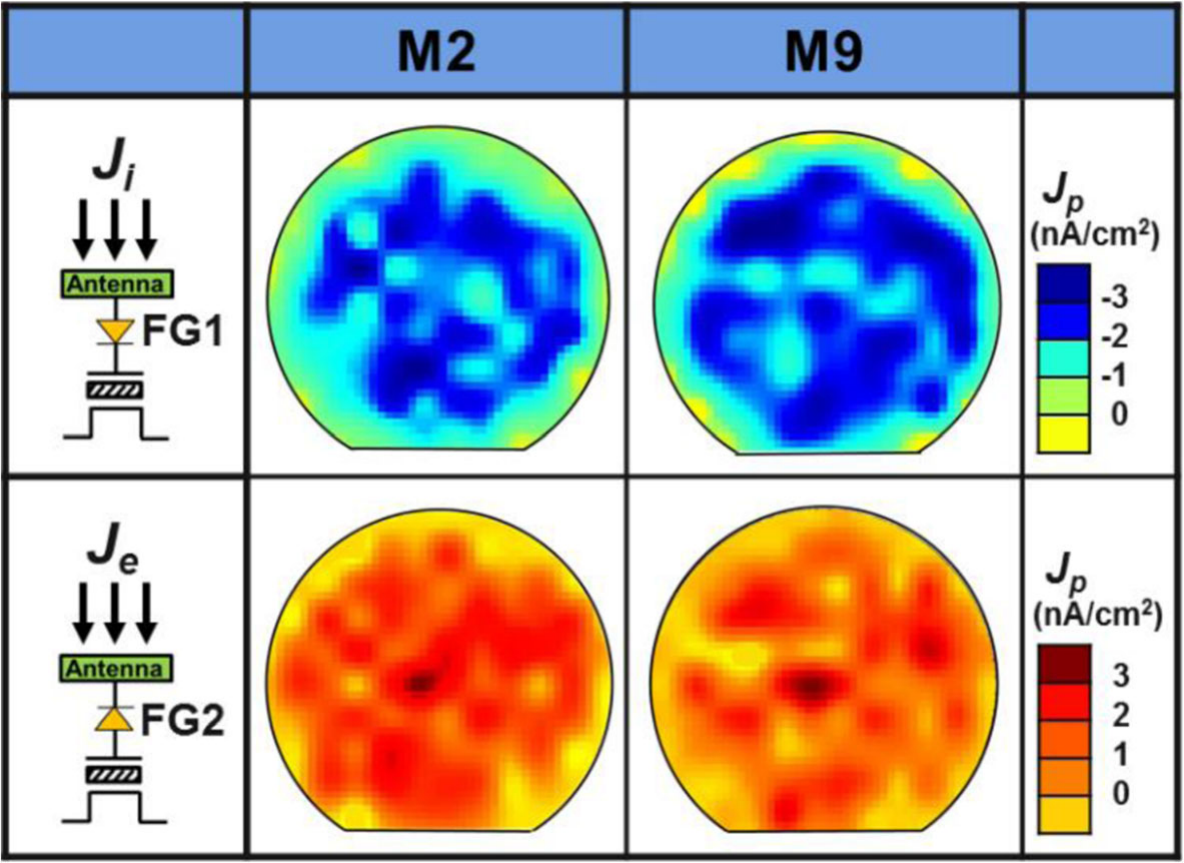
The projected electron and ion charging rate, *J*_e_(*x*,*y*) and *J*_i_(*x*,*y*) are obtained by the charge splitting recorders across the etching surface during metal 2 metal 9 formation

The distribution of positive and negative charging rates across a wafer during processing of the top (metal 9) and bottom metal (metal 2) layers are further compared in Fig. 8. It implies that charging on the antenna structure is more prominent at higher metal levels (metal 9), because on metal 9, its higher plasma energy causes *J*_p_ to be larger than *J*_p_ of metal 2 in terms of magnitude. Also, data suggests that both electron and ion charging rates peak around the center for both cases. As expected, dies closed to the center of the wafer experience high charging level, which can be attributed to the longer discharging path during plasma treatment. This location effect is found to be identical for both more electron and ion charging dominant conditions. The projected plasma charging rate, *J*_P_ (*x,y*), averaged over the formation of a single metal layer, metal 2 (M2) and metal 9 (M9), are further compared in Fig. 9. These wafer maps reveal that electron charging rate seems to be at a plateau except at the edge, while ion charging rates showed a higher variation in the middle section of the wafer. In the future, these wafer maps under different processing conditions are expected to provide insights to the plasma chamber, or further optimization guidelines by better compensating the charging effects.

### Antenna Ratio Effect

**Fig. 10:**
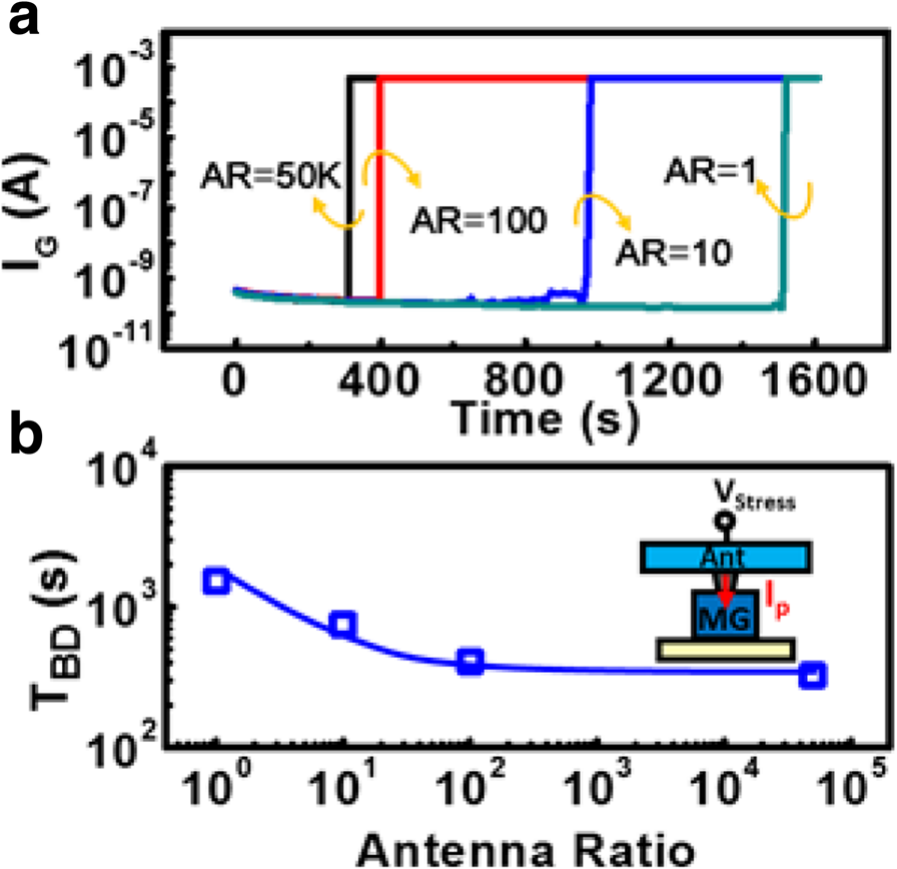
**a** The time-to-breakdown characteristics *I*_G_ vs time of the conventional PID detectors with the increasing antenna size. **b** *T*_BD_ decreases drastically as AR exceeds 1000

**Fig. 11:**
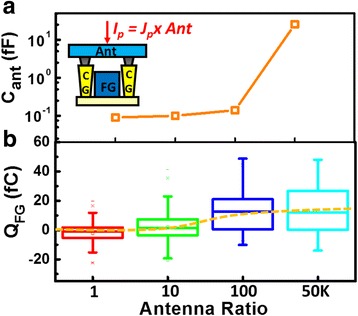
**a** As the capacitance of the antenna increases, *V*_CG_ becomes independent of the AR. **b** *Q*_FG_ saturates as the AR exceeds 100×

Traditional PID monitoring devices are typically designed to amplify the PID effect by connecting the transistor’s gates directly to a large antenna, evaluating the stress levels by increase of the total *Q*_P_ expected to be discharged through a small channel region [30, 31]. Antenna ratio (AR) is proportional to the stress current density through the gate dielectric during plasma processes [32]. Large *Q*_P_ on the antenna is known to induce latent damage and/or traps in the dielectric layer, which ultimately lead to reliability degradation [33]. As expected, higher AR on conventional FinFETs does significantly elevate the stress levels, causing a more severe T_BD_ degradation, namely, device failure within a shorter period of operation, see Fig. 10. On the other hand, in a CSIR, the plasma charging level recorded as the floating gate charge, *Q*_FG_, shows very little antenna effect. Namely, it does not respond to increasing antenna area, as revealed by the data summarized in Fig. 11.

In the new floating-gate-based CSIR, antenna ratio (AR) will affect the peak potential on the coupling gates during plasma charging. In scaled technologies, the parasitic capacitances on the connection and coupling structure are expected to reduce, leading to less AR sensitivity on the recording results. The reasons for leading to such an obvious difference to AR effect between CSIR and conventional detectors are as followed. In these floating gate recorders, the charge accumulated on the antenna, *Q*_P_ will not be discharged through the channel area. Increased *Q*_P_ raises *V*_CG_, leading to electron injection or ejection into/from the floating gates. As shown in the simulated capacitance in the Fig. 11a, the capacitance of the antenna, *C*_ant_, increases proportionally to antenna area, *A*_ant_. With the total charge current directly proportional to antenna area, increased AR in a CSIR will not affect the potential on the antenna. Measurement data reveal that the *Q*_FG_ level remains about the same for SCIR with AR exceeding 100×.

This feature not only saves test pattern area, but also enables finding *J*_P_ (*x*,*y*) with higher spatial resolution for studying patterning effect on PID. Besides, a detector with small antenna can facilitate the design of test patterns for understanding PID in middle-end of the line (MEOL) and contact processes.

Finally, performance summary of the new CSIR for monitoring PID in advanced BEOL FinFET process is summarized in Table 2. The sense range of the traditional detector is AR, while the sense range of the new in situ recorder is based on floating gate length. Further, the area of new in situ recorder can be very small. Most importantly, the new CSIR can provide the real-time feedback of plasma process and separate levels of ion charging and electron charging rate, independently.

**Table 2:** Performance summary of the new charge splitting in situ recorder (CSIR) for monitoring plasma charging damage in advanced FinFETs BEOL process

PID detector	Readings	Charge polarity separation	Read-time feedback	Sensitivity control	AR
Conventional	T_BD_	No	No	Antenna ratio (AR)	>10^3^
CSIR	Q_FG_	Yes	Yes	Coupling ratio (CR)	~10^2^

## Conclusions

A novel charge splitting in situ recorder (CSIR) for monitoring plasma-induced damage is first-time proposed and demonstrated. The CSIR provides a powerful tool for understanding electron charging and ion charging rates in a plasma chamber simultaneously. Wafer maps can facilitate further study between the correlation to device reliability and these individual charging effects.
