# Correction to: Charge Splitting In Situ Recorder (CSIR) for Real-Time Examination of Plasma Charging Effect in FinFET BEOL Processes

**DOI:** 10.1186/s11671-017-2336-x

**Published:** 2017-11-08

**Authors:** Yi-Pei Tsai, Ting-Huan Hsieh, Chrong Jung Lin, Ya-Chin King

**Affiliations:** National Tsing Hua University, Hsinchu, Taiwan

## Correction

In the original publication [1] Fig. 3 was presented incorrect. The correct additional file has been included with this erratum and the original article has been updated to rectify this error.

**Fig. 3:**
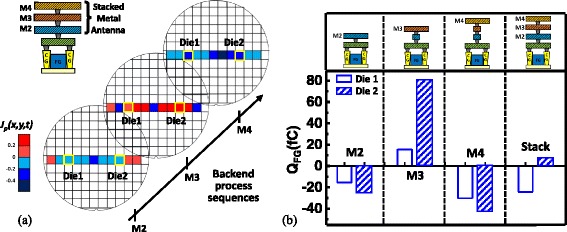
**a** The plasma charging effect for the different metal layers varies on different locations across the wafer. **b** The positive and negative charges may compensate each other in the stacked metal layers
